# The Economics of Innovation in the Prosthetic and Orthotics Industry

**DOI:** 10.33137/cpoj.v4i2.35203

**Published:** 2021-09-21

**Authors:** J. Andrysek

**Affiliations:** 1 Bloorview Research Institute, Holland Bloorview Kids Rehabilitation Hospital, Toronto, Canada.; 2 Institute of Biomedical Engineering, Faculty of Applied Science and Engineering, University of Toronto, Toronto, Canada.

**Keywords:** Innovation, Health care, Economics, Cost-effectiveness, Effectiveness, Rehabilitation, Assistive devices, Technology

## Abstract

Innovation is an important part of the prosthetic and orthotics (P&O) industry. Innovation has the potential to improve health care services and outcomes, however, it can also be a burden to the system if misdirected. This paper explores the interaction of innovation and economics within the P&O industry, focusing on its current state and future opportunities. Technological advancement, industry competition and pursuit of better patient outcomes drive innovation, while challenges in ensuring better P&O health care include lagging clinical evidence, limited access to data, and existing funding structures. There exists a greater need for inclusive models and frameworks for rehabilitation care, that focus on the use of appropriate technology as supported by research and evidence of effectiveness and cost-effectiveness. Additionally, innovative business models based on social entrepreneurism could open access to untapped and underserved markets and provide greater access to assistive technology.

## BACKGROUND

One can appreciate the technological advancement within the prosthetic and orthotic (P&O) industry. Advanced materials, body interfaces, and control systems are just some of the innovations making it possible for persons with severe disabilities such as limb loss or impairments to regain physical function and their lives. The advancement in P&O technology investment into product innovation, presumably a result of the competitive nature of the industry and desire for constant improvement of existing technologies, patient care and outcomes. Advancement also responds to and affects economics, both at the health care level where technological interventions are utilized, and at the market level where products are developed and commercialized. So, what drives innovation and focuses product developers and manufacturers to tackle certain areas of technological advancements over others? Are these driving forces achieving the desired goals in terms of rehabilitation care and outcomes, and if not, what is missing and what can be done? Is the focus to restore the most amount of human function possible for an individual or is it to take into account economic factors and try to restore the most amount of human function of the global population that suffers from a condition? This paper aims to explore the interaction of innovation and economics of the P&O industry, focusing on the drivers of innovation including competition, technological advancement, and betterment of patient outcomes, and also the challenges including lagging clinical and cost-effectiveness evidence, research biases, existing funding structures, and the need for more inclusive models and frameworks for rehabilitation care.

## INDUSTRY OVERVIEW

Prosthetic and orthotic technologies are part of a large and rapidly growing medical device industry valued at over US $8 billion in Canada, and over US $150 billion in the United States.^1,2^ The global market for prosthetics and orthotics is estimated at US $6 billion with an annual growth of nearly 5% - attributed largely to a growing population in need of such treatments.^3^ P&O treatments (i.e. prosthetic and orthotic devices) commonly cost thousands to tens of thousands of dollars. These costs are reoccurring every several years as devices reach the end of their lifecycle, or the patient's condition changes affecting fit and comfort, function, or desired rehabilitation goals. The process of P&O device procurement involves a number of stakeholders, including clinicians that determine and provide the patient with the devices, funders and insurers to cover portions of the cost, and the patient with their particular rehabilitation goals. All these parties typically have some say in determining which particular device or treatment is provided. It is essential that device developers and manufacturers understand these intricacies of the health care systems when developing new products to ensure that their devices can have both a viable market and provide large scale impact on the affected populations.

## TECHNOLOGY DEVELOPMENT PROCESS IN P&O

The P&O device industry is highly competitive. It is led by several large international corporations, each providing a variety of products. Outside of commonly defined categories of products designed for a particular patient demographic or health condition, understanding the technical nuances and product differentiation can present challenges in the clinical decision-making process. Hence, competing manufacturers strive for transformational advances to gain competitive advantage. Such advances are often closely tied to technological advancements in other industries. For example, a leap forward in terms of strong, flexible and lightweight materials to advance foot, socket and brace designs occurred with the introduction of composites such as carbon fibre laminates in the 1980s. The 90's which brought more powerful mobile computing, enabled the commercialization of microprocessor controlled prosthetic components such as the Intelligent Prosthesis from Blatchford. In the 2000's, advancements in power storage (i.e. high-density batteries) along with more powerful actuation systems have laid the foundation for powered lower-limb prostheses and exoskeletons. Therefore, the adoption and adaption of scientific and technological advancements from other industries is one key driver of innovation in P&O, providing a competitive advantage to companies that invest heavily into research and development, and patients with improved rehabilitation care via access to more advanced assistive devices.

The development of advanced P&O products, does not in itself ensure better health care and rehabilitation outcomes. Developing useful new technology requires a design process that carefully considers and addresses the needs of the relevant stakeholders (end-user, clinicians, funders and insurers etc.). As with most medical devices, the development and commercialization of P&O technology is a complex and resource intensive process. A new P&O technology can go through many design iterations informed by modeling and empirical testing, prior to finding its place in the marketplace. R&D is typically facilitated via both academic and industry driven research, or a combination of both. Government research grants typically help to fund early exploratory aspects of the R&D process. These can include industry-partnered programs which can leverage industry funds with grant money, or R&D tax credits for a company. Company resources or business investment are usually needed for later-stage development, market testing and commercialization.^4^ Established companies will typically resort to internal resources to fund product development and commercialization, while start-ups may need to raise financing through external investors (for example angel investors). Occasionally, projects may in part be supported by donors or foundation grants. At the early stages, stakeholder (user, practitioner, funder, industry) involvement is essential to define the criteria for the design. Methods such as the Quality Function Deployment and the House of Quality can be used to organize and prioritize design criteria.^5,6^ Criteria can change as the development progresses and should therefore be regularly reassessed. As part of an iterative design process, simulations and prototypes can enable testing to determine how well the design works and meets the desired criteria. While these steps are essential to inform the development of the product ahead of commercialization, establishment of clinical evidence about the efficacy and effectiveness typically happens once a product is on the market.

## QUALITY, EFFECTIVENESS, VALIDITY

The effectiveness, and more importantly cost-effectiveness, of medical interventions requires empirical clinical data, typically in the form of research-grant-funded clinical trials. Clinical research informs which treatments are most useful in decreasing the burden of a health condition, and in the case of P&O decreasing the effects of the disability and improving quality of life. Based on Health Canada and its much larger United States counterpart the Food, Drug, and Cosmetic Act (FDA), prosthetics and orthotics are classified as ‘low risk’ medical devices. Similarly, the sale of P&O devices in Europe is subject to CE designation which is an administrative marking that indicates conformity with health, safety, and environmental protection standards. However, none of the regulatory bodies necessitate that P&O devices undergo formal testing for safety or effectiveness. Bodies such as the International Organization for Standardization (ISO) have developed standards, for example to test the strength of lower-limb prosthetic components (e.g. ISO10328), however, these are not mandatory. Hence, unlike other interventions including drugs, P&O manufacturers are not required to provide evidence about the safety or effectiveness of their products. However, such data can play an essential role in the clinical decision-making processes, allowing for more informed clinical decisions about the suitability of the treatment options available. Empirical data can also play an important role in the establishment of new reimbursement codes (i.e. L-codes in the United States), thus making new devices attainable. Studies demonstrating effectiveness, can further play an important role in marketing, particularly to P&O practitioners who in large part lead the purchase decision-making process. Cost-effectiveness analyses, which compare treatments and their relative costs and outcomes, enable funders, governments and health care professionals and institutions, to make informed decisions about the care that is provided within funding constrained health care systems.^7^

Unfortunately, the clinical evidence relating to most P&O interventions is lacking or is not of high quality. The highest evidence comes from meta-analyses of RCTs (Level 1), followed by at least one RCT (Level 2), quasi experimental designs (Level 3) and so on, and the majority of clinical research studies involving P&O interventions fall in the latter categories.^8^ The customization of aspects of the P&O treatments, heterogeneity of patient populations, broad range of technologies and products, as well as varying patient goals and outcome measures, are just some of the challenges in designing quality research studies. Methodological issues, such as the inability to apply double blinding to the intervention (for example, participants or prosthetists can not readily be blinded when testing prosthetic knees that have distinct function or instructions for use), introduce potential study biases, compromise the resulting evidence, and fail to fully uphold the accepted methodological standards of RCTs. Availability and ability to attain funding for clinical studies is also a significant challenge. A typical multi-year RCT can easily cost $500,000 or more. In P&O, the high cost of componentry can further increase required funding, and thus may not be viewed favourably by grant review committees and dismissed as not being an effective use of grant/tax-payer monies.

In Canada, funding for clinical trials would typically be sought from Canadian Institutes of Health Research (CIHR). CIHR grant applications are extremely competitive, and P&O is up against a broad range of other healthcare priorities, such as finding the cure for cancer, which has relevance to a much larger part of our population. Hence, P&O researchers commonly resort to applying for smaller grants, which limits the types of studies and quality of clinical evidence. Possibly due to aforementioned restrictions in accessing traditional grant funding, many studies evaluating P&O products are sponsored by companies selling the products. While this may be a means for establishing studies and providing at least some evidence, the data may be potentially susceptible to biases, or at least a perception thereof.^9^ There is little incentive for a company to disseminate results that do not demonstrate their product to be superior and bring the anticipated benefits. With higher level studies including registered clinical trials, there exist greater oversights for unbiased reporting of results; unfortunately, in P&O such studies are not common. Another major challenge is the latency in establishing clinical evidence. For example, microprocessor knee joints were introduced to the market in the mid 1990's, however, to this date, evidence is based on not one RCT.^10^ These limitations make it challenging for stakeholders to know which innovations truly serve the needs of the patient and health care system.

While many innovations improve our lives, some may bring undesired adversities, complexities and disenchantment. For example, despite the advancements in electro-mechanical prostheses including myoelectric hands, much simpler purely mechanical body-powered devices are still highly utilized in clinical care.^11^ Some of this is attributable to the high cost of myoelectric devices, but also likely their limited function and utility. With most health care expenditures being contained, innovation can be a major inflationary factor, and the high cost of P&O treatments requires especial consideration of cost-effectiveness. For example, while microprocessor knee joints are shown to provide benefits including the reduction of falls, their acquisition and maintenance costs are significantly higher than their mechanical counterparts.^7,10^ Many public health care systems do not fully cover the cost of P&O devices, and especially those at the higher end of technological sophistication. As such, significant inequalities exist in terms of access to modern innovations typically costing significantly more than the status quo. These discrepancies are apparent in places like Canada, where clinical provision of high-end P&O technology is far from universal.^12^ Even more striking, 85% of the world's disabled population lack access to even the most basic P&O interventions.^13^ While this immense problem is not solely due to the lack of cost-effective devices, it nevertheless suggests that our efforts to innovate may be to some extent misguided.^14^

## FUTURE DIRECTIONS

Innovation is most commonly associated with the advancement of sophisticated P&O technology, however, could and should more of our energies be directed elsewhere? Should we, for example, focus on the development of technology that is simpler, more cost-effective, and still adequately functional? Such an approach could potentially achieve greater equality and access to P&O devices. Perhaps the focus should be less on the promotion of sophisticated devices, and more at taking measures to study and ensure that a device is appropriate for its application. For this, research needs to step beyond simply assessing select aspects of device performance under laboratory conditions, and towards more real-life and comprehensive assessments that capture what is truly important to the person using the device, as well as the health care system.^15^ Greater understanding yet, is needed in evaluating the quality of life outcomes and cost-effectiveness of P&O care and devices. Finally, a greater understanding, which could be obtained via research, is needed about the factors including technological, economic, healthcare, cultural, demographic, regulatory aspects, that drive the care and devices that are provided. Currently, the available information is anecdotal at best. Establishment of data in these respects could help in the development of new frameworks and approaches for the provision of assistive technology, concomitantly targeting greater access, better outcomes, and higher efficiency.^16^

Innovative approaches to service and device delivery have had significant impact in other areas of health care to provide greater access to quality care. In the 1980's, for example, the high cost of implantable intraocular lenses (IOL) restricted access to cataract surgery amongst the poor in India. This led to the development of new manufacturing facilities, capable of producing IOLs at a small fraction of the market price.^17^ Lower price not only enables greater access, but also increases sales, which in turn increases manufacturing output and efficiencies. Innovative new industry entrants can therefore disrupt the competition and in this particular case the IOL venture was able to significantly increase access to IOL and cataracts surgery. The innovative approach established in the IOL industry also included a hybrid business model, focused on sustainable business operations while in parallel serving a social mission to provide greater access. Similar models have been demonstrated in P&O, however greater initiative is needed by companies, for example, LegWorks Inc, and organizations with a strong presence and influence in the market.^18^ Alternatively, greater focus on training the next generation of social entrepreneurs with a focus on P&O, could help to establish innovative and more inclusive models and frameworks for rehabilitation care to advance promising ongoing efforts involving various organizations and partnerships.^16,19-21^ Moreover, serving to the needs of the less-resourced markets, as hybrid models aim to do, could in turn drive the development of cost-effective and affordable P&O devices, as opposed to the current status quo where low cost devices are typically of low function and in some cases lower quality.

## CALL TO ACTION

Much like the human body, the P&O industry is an amazingly complex system. Technological ingenuity has allowed assistive devices to successfully restore the body's function for those that have access to such technology. The P&O industry can and should take certain steps to ensure that rehabilitation care reaches all those that need it. Organizations such as the International Society of Prosthetics and Orthotics (ISPO) need to take a leadership in identifying and defining P&O priorities through engagement and consensus of stakeholders and industry experts many of whom are members of ISPO. In this way ISPO and other professional organizations can help to inform and advocate around the pressing issues towards a focused action plan. Consensus of priorities will provide a stronger and more justifiable foundation for researcher to build competitive funding applications. Recent initiatives and reports developed by the Clinton Health Access Initiative (CHAI) under the AT2030 programme in support of the AT-scale Strategy are an excellent example of such an effort. For the P&O industry specifically, there now exists a comprehensive globally relevant narrative identifying the barriers and exploring ways for better P&O service delivery.^16^ Such works could similarly help to structure and prioritize research and development efforts. As such, a greater understanding of the workings of the P&O industry could help to identify the gaps and opportunities to truly advance P&O care. Focusing a greater part of innovation on simpler technology and using empirically derived evidence to inform its use in clinical care, could also help ensure that appropriate technology is utilized. There needs to be more incentive for companies in this regard. Internally, companies can decide to uphold a greater focus on what might be less profitable but more impactful projects and products, for example by having a social for-profit business structure focused on developing and providing affordable, appropriate and high-quality prosthetic components within both high- and low-income countries.

Innovative business models based on social entrepreneurism could open access to untapped and underserved markets, thus making social entrepreneurism a viable, sustainable and potentially profitable approach for companies. It may be possible to adapt and scale the examples of IOL and LegWorks Inc as described above. However, governments also have a role to play since to an extent they dictate what products are lucrative to develop and sell in the presiding healthcare ecosystems. In the United States, the reimbursement codes and categories are largely based on the technical features of a device, rather than a metric of performance. This is likely in part due to the fact that performance measures require clinical evidence, which as described previously is greatly lacking in P&O. Additionally, in markets such as the United States, existing reimbursement systems favour high-end devices which yield greater margins for the clinics and companies selling components. Hence governments play an important role in managing these aspects of the medical device industry. Government also provides research funding and sets scientific priorities.

A greater level of communication and coordination is needed amongst different industry stakeholders, including patients, clinicians, healthcare institutions, professional organizations, companies, academia, and government to identify and tackle the key priorities, such as the generation of data relating to cost-effectiveness for informing governmental policy, and establishing the proper funding systems and rehabilitation health care services. Such efforts need to be driven by organizations that are comprised of representation from all of the stakeholders, such as ISPO at the international level, and locally with organizations such as Orthotics Prosthetics Canada (OPC) or American Orthotic & Prosthetic Association (AOPA) in the United States. For the common goal of all of the stakeholders in the P&O industry should be clear, and that is to provide equitable access to P&O care, and to enable individuals to successfully rehabilitate.

## DECLARATION OF CONFLICTING INTERESTS

Jan Andrysek is a co-founder and active member of LegWorks Inc. He is also a member of Exceed Worldwide and ISPO.

## SOURCES OF SUPPORT

None.

## AUTHOR SCIENTIFIC BIOGRAPHY

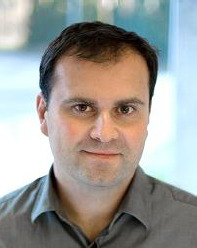



**Dr. Jan Andrysek** is a Senior Scientist at the Bloorview Research Institute of Holland Bloorview Kids Rehabilitation hospital. He is also an Associate Professor at the Institute of Biomedical Engineering, University of Toronto. His research program focuses on the development of treatments and assistive technologies for children and youth with disabilities. Specific areas of study include prosthetic and orthotic limb control, bio sensing and biofeedback systems, and instruments to measure assistive-technology-facilitated mobility and physical activity in real-life environments. Current research is also focused on understanding the global need for prosthetic technology, and impact on mobility, physical function, and quality of life. He is the recipient of awards including the 2017 Ontario Profession Engineers Engineering Medal for Research and Development, Clifford Chadderton Award for Prosthetics and Orthotics Research, and first price at the 2015 Accessibility Innovation Showcase Tech Pitch Competition sponsored by the Government of Ontario. In 2019 Dr. Andrysek was elected an American Institute for Medical and Biological Engineering (AIMBE) fellow. Dr. Andrysek is also the co-founder and Chief Scientific Officer at Legworks Inc., a social for-profit enterprise focused on improving prosthetic technologies and care for individuals with amputations worldwide.
